# Impact of crestal and subcrestal implant placement in peri-implant bone: A prospective comparative study

**DOI:** 10.4317/medoral.20747

**Published:** 2015-11-30

**Authors:** Hilario Pellicer-Chover, María Peñarrocha-Diago, David Peñarrocha-Oltra, Sonia Gomar-Vercher, Rubén Agustín-Panadero, Miguel Peñarrocha-Diago

**Affiliations:** 1 DDS. Master in Oral Surgery and Implant Dentistry, Faculty of Medicine and Dentistry, University of Valencia, Spain; 2 MD, PhD, DDS. Professor of Oral surgery, Director of Master’s Program in Oral Surgery and Implant Dentistry, Faculty of Medicine and Dentistry, University of Valencia, Spain; 3 PhD, DDS. Associate professor, Department of Stomatology, Valencia University Medical and Dental School, Valencia, Spain; 4 PhD, DDS. Private practice, Valencia, Spain; 5 MD, PhD. Chairman of Oral Surgery and Director of the Master in Oral Surgery and Implant Dentistry, Faculty of Medicine and Dentistry, University of Valencia, Spain

## Abstract

**Background:**

To assess the influence of the crestal or subcrestal placement of implants upon peri-implant bone loss over 12 months of follow-up.

**Material and Methods:**

Twenty-six patients with a single hopeless tooth were recruited in the Oral Surgery Unit (Valencia University, Valencia, Spain). The patients were randomized into two treatment groups: group A (implants placed at crestal level) or group B (implants placed at subcrestal level). Control visits were conducted by a trained clinician at the time of implant placement and 12 months after loading. A previously established standard protocol was used to compile general data on all patients (sex and age, implant length and diameter, and brushing frequency). Implant success rate, peri-implant bone loss and the treatment of the exposed implant surface were studied. The level of statistical significance was defined as 5% (α=0.05).

**Results:**

Twenty-three patients (8 males and 15 females, mean age 49.8±11.6 years, range 28-75 years) were included in the final data analyses, while three were excluded. All the included subjects were nonsmokers with a brushing frequency of up to twice a day in 85.7% of the cases. The 23 implants comprised 10 crestal implants and 13 subcrestal implants. After implant placement, the mean bone position with respect to the implant platform in group A was 0.0 mm versus 2.16±0.88 mm in group B. After 12 months of follow-up, the mean bone positions were -0.06±1.11 mm and 0.95±1.50 mm, respectively - this representing a bone loss of 0.06±1.11 mm in the case of the crestal implants and of 1.22±1.06 mm in the case of the subcrestal implants (*p*=0.014). Four crestal implants and 5 subcrestal implants presented peri-implant bone levels below the platform, leaving a mean exposed treated surface of 1.13 mm and 0.57 mm, respectively. The implant osseointegration success rate at 12 months was 100% in both groups.

**Conclusions:**

Within the limitations of this study, bone loss was found to be greater in the case of the subcrestal implants, though from the clinical perspective these implants presented bone levels above the implant platform after 12 months of follow-up.

**Key words:**Immediate implants, tooth extraction, dental implants, single-tooth, crestal bone, placement level.

## Introduction

The connecting line between implant and abutment, the so-called microgap, has been intensely investigated during the last 10 years. This micro gap has been cited as one of the factors capable of influencing peri-implant bone resorption in conjunction with other factors such as surgical trauma, the establishment of biological width, implant design and implant positioning (1-3). The preservation of peri-implant bone is an important factor for success. The quantity and quality of the bone surrounding an implant affects implant osseointegration, influences the shape and contour of the overlying soft tissues, which are important for the esthetic outcome of treatment (4), and it has been reported that if an implant with a rough surface is exposed to the oral cavity, a greater amount of plaque, leading to perimucositis and peri-implantitis, may be present (5). The occurrence of peri-implantitis around implants with roughened surfaces is likely to be even higher, since it was observed that statistically significantly more peri-implantitis occurred at 3 years of loading around implants with roughened surfaces when compared to turned implants (6).

Branemark *et al*. (7) recommended surgical implant countersinking below the bone crest, which prevents implant exposure during bone remodeling. Well-documented long-term clinical studies with these systems have also revealed highly predictable outcomes (8-10). In contrast, several studies have shown the absence of a micro gap at or below the alveolar crest level in non-submerged implant systems to result in less peri-implant marginal bone loss than with submerged implant systems (11,12). Furthermore, apical positioning of the implants did not influence ridge loss or the position of the peri-implant soft tissue margin (13). On the other hand, subcrestal implant placement has recently been associated to increased marginal bone loss (14), and Hammerle *et al*. had already concluded that such an approach was not to be recommended (9).

Such increased loss may be caused by bacterial colonization of the micro gap present in the fixture-abutment junction (15). The inside of the connection has a low oxygen concentration and is away from the inflammatory defensive response of the peri-implant tissues, so it is an ideal environment for anaerobic bacteria (16). The potential colonization through the micro-gap is related to multi factorial conditions, including the precision fit between the components which is associated with the implant system design. A recent systematic review (17) claimed the superiority of conical connections in seal performance, microgap formation, torque maintenance and abutment stability.

The aim of this study was to assess the influence of the crestal or subcrestal placement of implants upon peri-implant bone loss over 12 months of follow-up.

## Material and Methods

- Patient screening and recruitment

Patients with a single hopeless tooth were recruited (Oral Surgery Unit, Valencia University, Valencia, Spain). The research was performed following the principles of the Declaration of Helsinki on research involving human beings and the study design was approved by the ethical review board of the University of Valencia (Ref: H1365580155510).  Table 1 specifies the inclusion and exclusion criteria. Patients who met the criteria and agreed to participate in the study were asked to read, understand, ask questions, and sign an informed consent form. The study was conducted from December 2012 to August 2014.

**Table 1:**
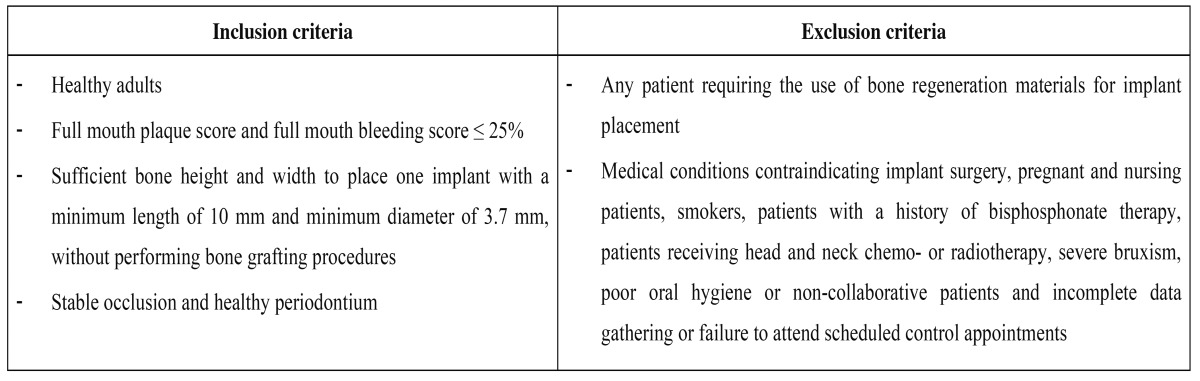
Patient inclusion and exclusion criteria.

- Preoperative procedure

Upper and lower alginate impressions were taken from each patient for planning and fabricating measurement stents and surgical guides, all patients received rigorous oral hygiene and were given instructions for improving and maintaining oral hygiene at home. Extraction of teeth was done with great care; in the case of multiple root teeth, dental sectioning was performed and the roots were extracted separately, respecting the alveolar walls (especially the vestibular wall). The patients were instructed to wear a removable, tooth-supported provisional prosthesis during the healing phase (only in the esthetic zone).

**Figure 1:**
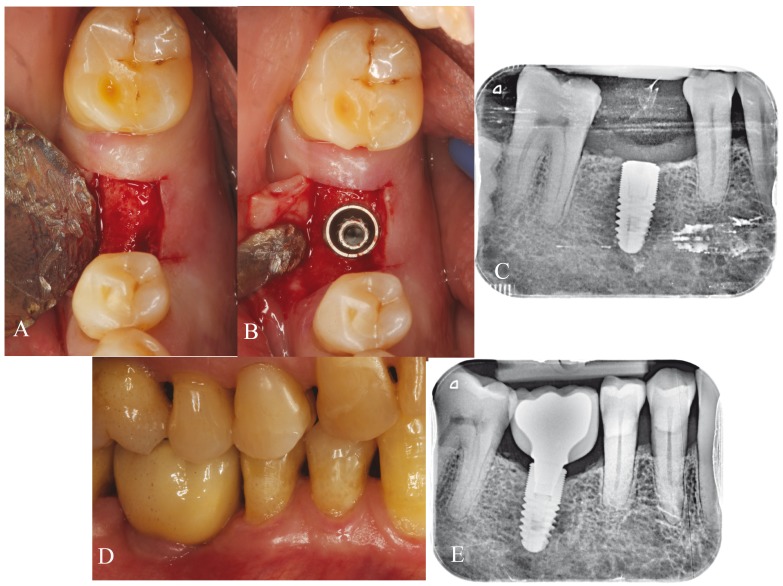
Crestal group. A) Interdental space in the fourth quadrant. B) Implant placement at crestal level. C) Periapical X-ray view of the implant at the time of placement. D) Screwed definitive prosthesis. E) Periapical X-ray view of the implant after 12 months of follow-up.

**Figure 2:**
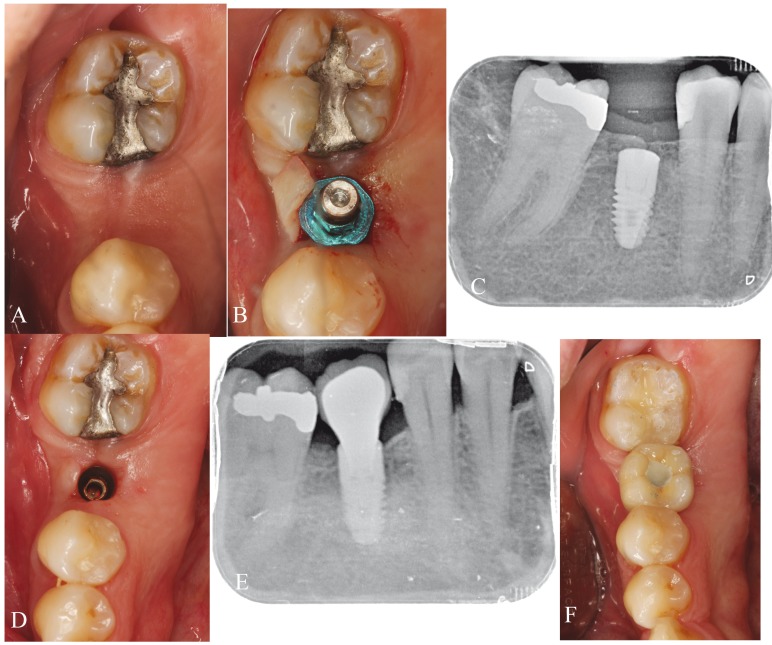
Subcrestal group. A) Interdental space in the fourth quadrant. B) Implant placement at subcrestal level. C) Periapical X-ray view of the implant at the time of placement. D) Healed gingiva after three months. E) Periapical X-ray view of the implant after 12 months of follow-up. F) Screwed definitive prosthesis.

After three months of tooth socket healing, each patient was randomized to one of two treatment regimens: group A (all implants were placed at crestal level) (Fig. 1) or group B (all implants were placed at subcrestal level) (Fig. 2). Random assignment was performed by a professional statistician using pre-defined randomization tables. A balanced random permuted-block approach was used to prepare the randomization tables in order to avoid unequal balance between the two treatment groups.

- Surgical procedure

All surgeries were performed under local anesthesia (4% articaine with 1:100.000 adrenalin [Inibsa®, Lliça de Vall, Barcelona, Spain]). In all maxillary cases drills and osteotomes were used in combination to prepare the implant beds when the operator (MP) sensed that bone density was low.

The implants used in the present study were Mozo-Grau® implants, presenting a neck design with micro threads, treated surface, internal connection, and platform switching (Inhex®, Mozo-Grau, S.L. Valladolid, Spain), following the manufacturer’s placement instructions, and all patients were treated following a two-step procedure. After implant placement and suturing, each patient received 500 mg of amoxicillin (Clamoxyl®, GlaxoSmithKline, Madrid, Spain) three times daily for 7 days, 600 mg of ibuprofen (Bexistar®, Laboratorio Bacino, Barcelona, Spain) to be taken as needed, and a 0.12% chlorhexidine mouthwash (GUM®, John O. Butler/Sunstar, Chicago, IL, USA) for use twice daily during two weeks. Gentle brushing with a chlorhexidine toothpaste was also recommended. Sutures were removed 8-10 days after surgery. Prosthetic loading in the maxilla was carried out after 8-10 weeks following implant placement, and after 6-8 weeks in the case of the mandible.

The same lab technician fabricated all of the restorations. Crowns screwed to the Morse cone internal connection of the implant were prepared. All the structures were made of chromium-cobalt and CAD-CAM drilled (Bio-CAM, Mozo-Grau, S.L., Valladolid, Spain) and a feldspathic ceramic veneering [IPS d.SIGN, Ivoclar Vivadent, Schaan, Liechtenstein] was used. All screws were tightened with a torque of 30 Ncm according to the manufacturer’s specifications. The access hole of the screw-retained crowns was closed with a teflon pellet and a hybrid resin composite [Tetric-Ceram, Ivoclar Vivadent, Schaan, Liechtenstein].

- Measurements

A previously established standard protocol was used to compile the following data on all patients: sex, age (at implant placement), implant length, implant diameter, and brushing frequency.

Two trained clinicians (HP and DP) worked together to interpret the radiographs corresponding to the two groups in a similar manner, at the following time points: at the time of implant placement (T1) and 12 months after loading (T2). At each time point radiological evaluation was carried out with an XMIND intraoral system (Groupe Satelec-Pierre Rolland, Bordeaux, France) and an RVG intraoral digital receptor (Dürr Dental, Bietigheim-Bissingen, Germany). To reproduce the patient alignments, a rigid cross-arch bar was used with bite-registration material, and a Rinn XCP (Dentsply, Des Plaines, IL, USA) rod and ring were firmly attached to the bar and placed in contact with the X-ray cone. The receptor was held by a slot in the bar. Software-based measurements were made (in mm) of implant marginal bone loss. For measurement purposes, two visible and easily localized reference points were selected at the implant platform. A straight line was traced joining the two reference points and was taken to represent zero height. For the determination of bone loss, a perpendicular line was traced mesial and distal to the implant from zero height to contact with the bone. The difference between the value recorded at the time of placement and after 12 months of loading was used to calculate bone loss mesial and distal to the implant. The average between mesial and distal was selected as the bone loss for the fixation in question, expressed as a positive value if the peri-implant bone was located coronal to the implant shoulder.

The measurement of bone loss allowed us to establish the exposed treated surface of the implant, which was defined as either the absence or presence of exposed surface (with the magnitude in millimeters).

The definition of implant success was based on the clinical and radiographic criteria described by Buser*et al*. (18): 1) absence of clinically detectable implant mobility; 2) absence of pain or any subjective sensation; 3) absence of recurrent peri-implant infection; and 4) absence of persistent radiotransparency around the implant after 12 months of loading.

- Statistical analysis

In the inferential analysis we initially assessed the homogeneity of the crestal and subcrestal implant groups for the variables referred to the implant and surgical characteristics, based on the chi-squared test, Fisher exact test and non parametric Mann-Whitney U-test. A non parametric Bruner-Langer model was adopted for the longitudinal data, evaluating the effects of time and position, as well as the interaction between both, using non parametric analysis of variance (ANOVA). The level of statistical significance was defined as 5% (α=0.05).

## Results

A total of 26 patients were enrolled in the study. One patient suffered a facial dehiscence at the time of implant placement, and two patients did not return to complete the study. These three patients were excluded from the final data analyses. Twenty-three patients (8 males and 15 females) with a mean age of 49.8±11.6 years (range 28-75 years) were included in the final data analyses. The patient demographic characteristics are shown in  Table 2. The 23 implants corresponded to 10 crestal implants (group A) and 13 subcrestal implants (group B).

**Table 2:**
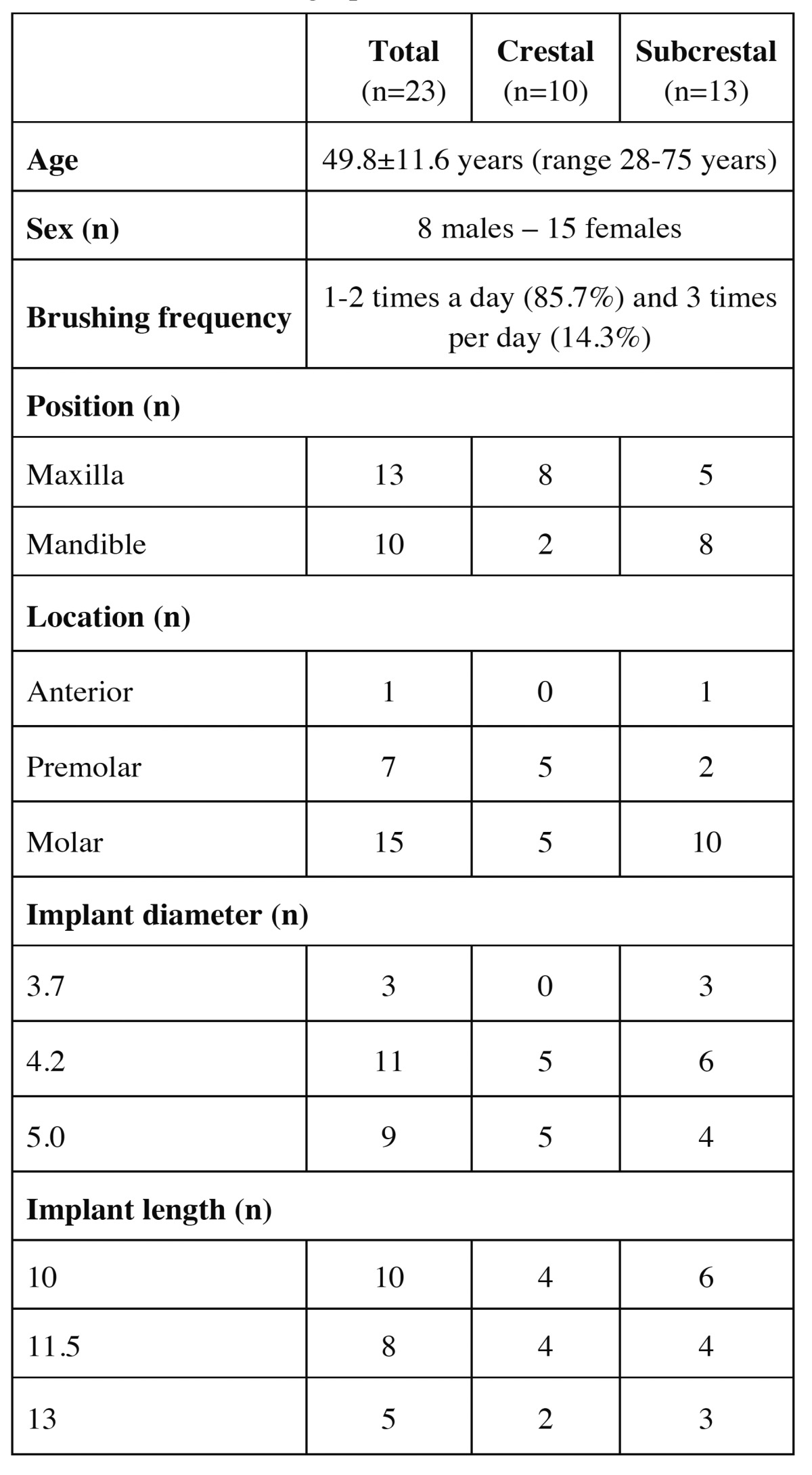
Patient demographic data.

**Figure 3:**
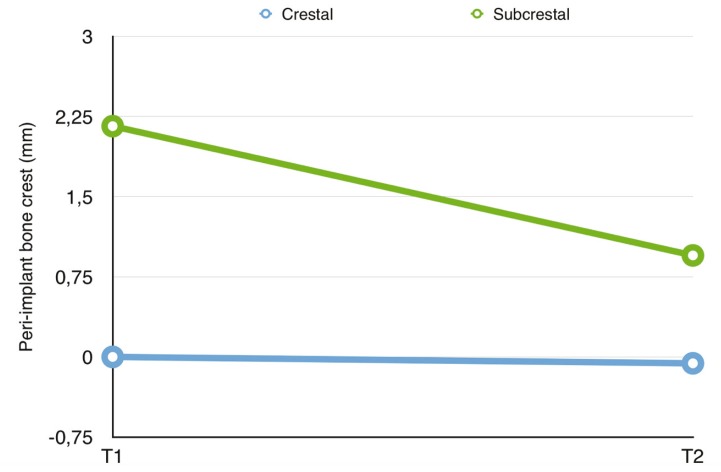
Evolution of the bone crest in the two groups at the different study timepoints.

 Table 3 shows the mesial and distal bone measurements of the crestal and subcrestal implants at the two time points considered (T1 and T2). Following implant placement (T1), the mean bone position with respect to the implant platform was 0.0 mm in group A versus 2.16±0.88 mm in group B. After 12 months of follow-up (T2), the mean bone positions were -0.06±1.11 mm and 0.95±1.50 mm, respectively – this representing a bone loss of 0.06±1.11 mm in the case of the crestal implants and of 1.22±1.06 mm in the case of the subcrestal implants (p=0.014)( Table 4). The crestal implants showed no significant variation from T1 to T2 (*p*=0.889), in contrast to the subcrestal implants (*p*=0.006). At T1 the differences in mean dimension between the two groups was significant (*p*<0.001), though by T2 the situation was seen to have homogenized (*p*=0.131) ( Table 4 and Fig. 3).

**Table 3:**
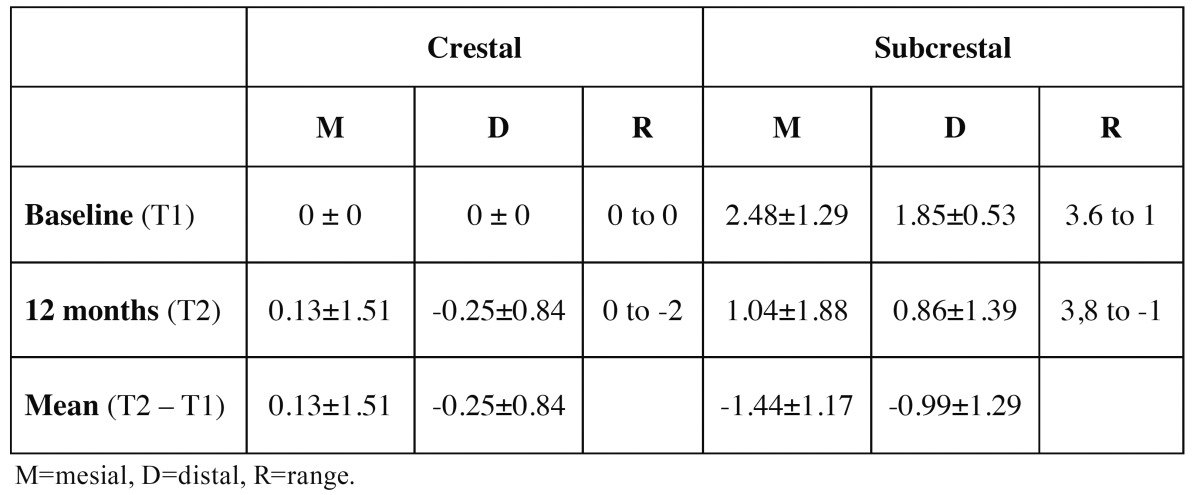
Mesial and distal bone measurements of the crestal and subcrestal implants at timepoints T1 and T2. The values are expressed in mm.

**Table 4:**
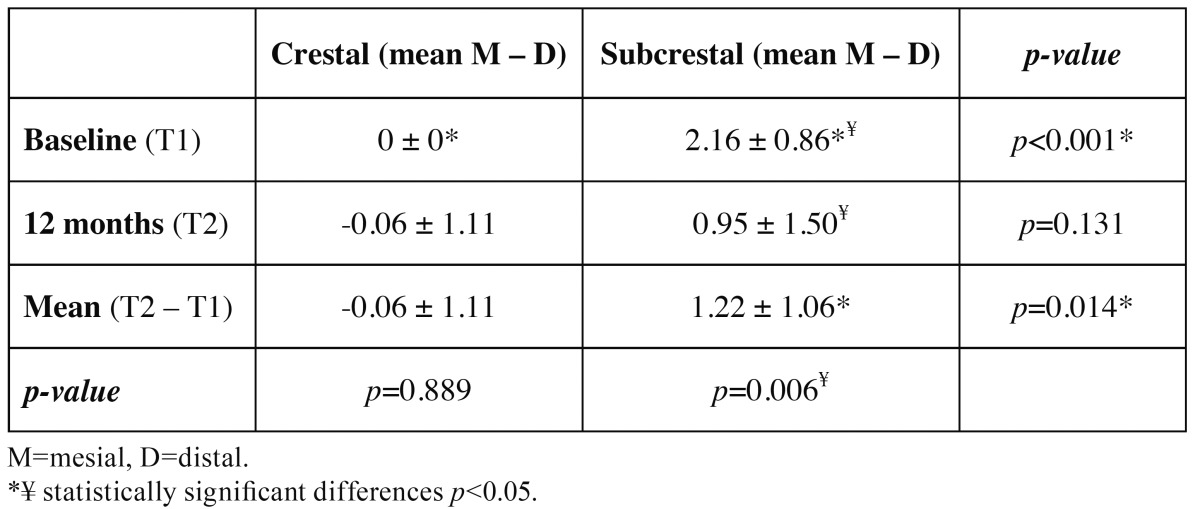
Mean bone measurements corresponding to the two study groups.

Six implants in group A maintained peri-implant bone at the level of the implant platform, while 8 implants in group B presented a peri-implant bone level an average of 1.96 mm (range 0.54-3.80 mm) above the platform. Therefore, 4 crestal and 5 subcrestal implants presented peri-implant bone levels below the platform, leaving a mean exposed treated surface of 1.13 mm (range 0.65 mm - 2 mm) and 0.57 mm (range 0.25 mm- 1.05 mm), respectively. The implant osseointegration success rate at 12 months was 100% in both groups.

## Discussion

The purpose of this study was to evaluate and compare the marginal bone loss and success of crestal and subcrestal implants rehabilitated with single crowns. Research involving implants placed subcrestally mainly comprise studies in animals (19-22), with fewer retrospective studies in humans (3,4,23,24). Only one prospective study has been found in the literature (25), involving a short follow-up of four months. Despite its reduced sample size of 23 patients and 23 implants and a short duration of follow-up (12 months), the present study aimed to contribute to evaluation and comparison of the marginal bone loss and success of implants placed at crestal and subcrestal level and rehabilitated with a single crown. The 23 consecutive patients were selected using strict, uniform criteria, and were treated by the same team of professionals using exactly the same procedures.

In the present study, peri-implant marginal bone loss was assessed from parallelized periapical radiographs, and was found to be 0.06±1.11 mm on average in group A versus 1.22±1.06 mm in group B, after 12 months of follow-up. These results are consistent with those obtained in other studies in humans (3,4,23-25), where the bone loss associated to implants placed at crestal and subcrestal level ranged from 0.5-1.5 mm and 0.08-1.78 mm, respectively. Albrektsson *et al*. (26) accepted 1 mm of peri-implant bone loss during the first year of function, followed by an annual loss of under 0.2 mm after the first year in service as criteria for implant success. This reestablishment of biological width may occur as result of micro movements at the implant–abutment interface (27), or may be due to bacterial migration and colonization of the micro gap on a screw-retained abutment (28). For this reason, some authors (9,11-14) consider that implant placement at subcrestal level may be deleterious for the maintenance of peri-implant bone, since it implies that the micro gap must lie below the peri-implant bone crest, which induces localized chronic inflammation (11). Piattelli *et al*. (16) histologic ally evaluated bone response associated to different microgap locations on the alveolar crest (implants inserted 1-2 mm above the alveolar crest, implants inserted at the level of the alveolar crest, and implants inserted 1-1.5 mm below the alveolar crest). They found that if the micro gap was moved coronally away from the alveolar crest, minimum bone loss and minimum inflammatory infiltration occurred.

This problem could be resolved with the introduction of the Morse taper internal connections (17,29), another recent review (30) concluded that, no implant system can currently provide a complete seal, occurring bacterial leakage irrespective of the type of connection. It also concluded that there is no evidence on the clinical significance of this microbial leakage. The Morse taper internal connection could reduce leakage to physiological and tolerable levels, which clinically constitutes success.

In our study, although crestal implant bone loss was smaller, the peri-implant bone starting point caused the implants to present a greater exposed treated surface (mean 1.13 mm) than in the case of the implants that had been placed at subcrestal level (mean 0.57 mm). Given that exposed treated surface of the implant could lead to complications in the peri-implant health (5), we suggest that subcrestal placement of the implants was found to be favorable, since peri-implant bone remained above the level of the implant platform or bone loss was minimal after 12 months of follow-up. From the clinical perspective, this could allow the maintenance of peri-implant bone for a longer period of follow-up, thereby counteracting the physiological bone remodeling observed over time, 

The implant osseointegration success rate at 12 months was 100% in both of our groups. We found few studies in humans (3,4,23-25), though with high success rates (100% in most cases). Only Koh *et al*. (25) reported one failure, resulting in a success rate of 95.8%, though the authors do not indicate whether the single failure corresponded to implant placement in the crestal or subcrestal position.

Human clinical studies involving longer follow-up periods and larger sample sizes are needed to determine the behavior of peri-implant bone in implants placed at subcrestal level, and to establish the effect of oral exposure of the treated surface of the implant. Within the limits of this prospective study, it could be suggested that subcrestal implants result in greater bone loss. However, from the clinical perspective these implants maintained peri-implant bone levels above the implant platform after 12 months of follow-up, which could compensate physiological bone remodeling.
